
JOURNAL INFORMATION
==============================
NLM Title Abbreviation: ScientificWorldJournal
Journal ID: ScientificWorldJournal
NLM Journal ID: 101131163
Journal ID: TSWJ

The Scientific World Journal

ISSN: 2356-6140
EISSN: 1537-744X
Publisher: Wiley

ARTICLE INFORMATION
==============================
PMCID: PMC5956388
PMID: 15349558
DOI: 10.1100/tsw.2004.89
Article ID: 790789
Article version: 1
Subjects: Original Report

54 year-old Female with Recurrent Bronchopneumonia and Tumor of the Left Kidney

Ubrig Burkhard
Roth Stephan
Hertle Lothar
Klinik und Poliklinik fuer Urologie WestfaelischeWilhelms-Universitaet Muenster, Albert-Schweitzer-Str. 33, 48149 Muenster, Germany
Academic Editor: Anthony Atala

Publication date: 2004
Electronic publication date: 2004 Jun 7
Volume: 4
First page: 353
Last page: 356
Copyright: Copyright © 2004 Burkhard Ubrig et al.
Copyright year: 2004
License: This is an open access article distributed under the Creative Commons Attribution License, which permits unrestricted use, distribution, and reproduction in any medium, provided the original work is properly cited.
License URL: https://creativecommons.org/licenses/by/3.0/

==============================
Original Report
*TheScientificWorldJOURNAL* (2004) 4 (S1), 353–356
ISSN 1537-744X; DOI 10.1100/tsw.2004.89

### 54 year-old Female with Recurrent Bronchopneumonia and Tumor of the Left Kidney

Burkhard Ubrig, MD, Stephan Roth, MD, and Lothar Hertle, MD
*Klinik und Poliklinik fuer Urologie Westfaelische Wilhelms-Universitaet Muenster, Albert-Schweitzer-Str. 33, 48149 Muenster, Germany*

*Previously published in the Digital Urology Journal*

**DOMAIN**: urology

### PATIENT HISTORY

- 54 year-old female
- Recurrent cystitis and pyelonephritis since 13 years, nephrolithiasis in both kidneys for more than 10 years, currently no urinary frequency, no dysuria
- Since 2 years: recurrent bronchopneumonias of unknown origin
- Chronic fatigue, persistent coughing

### PREVIOUS MEDICAL HISTORY

The patient has no other urologic disease. Recurrent bronchopneumonias during the last 24 months

### MEDICATIONS

Pipemidic acid 200 mg twice daily since 5 years

### PHYSICAL EXAMINATION

Obesity, no tenderness of the flank or the abdomen, no fever, no other abnormalities

### PRESENTING LABS AND IMAGING STUDIES

a. *Blood/Serum:* ESR (1 minute): 90 mm; RBC, WBC, serum-CRP, -creatinine,-BUN, -electrolytes, -ALT, -AST, -g-GT, -Bilirubin and -LDH within normal ranges
b. *Urine Sediment:* no hematuria, no leukocyturia, no other abnomalities
c. *Urine Culture:* negative d. *Urine Testing for TB:* first MSU of the morning (3 times): no acid-fast rod-shaped bacilli on microscopy, TB Test negative, TB culture negative 3 times
e. *Chest-radiographs:* solitary round opacity of 3 cm in diameter in the caudal lobe of the left lung
f. *Intravenous Urogram (IVU)(Fig.1):* Left kidney: extensive nephrolithiasis especially of the upper pole. Right kidney: duplicated unit, 3 calculi smaller than 5 mm.

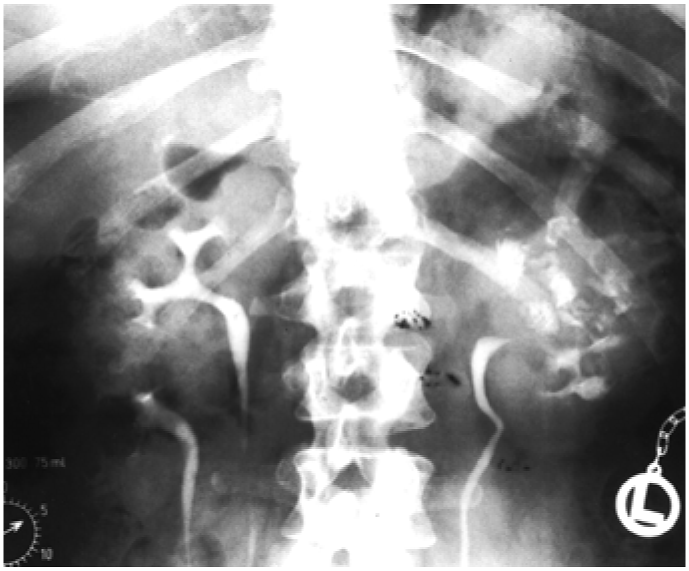

**FIGURE 1**

g. *Retrograde Pyelography of the Left Kidney:* The upper calyces of the left kidney are dilated; no fistula(Fig.2)
h. *CT-scan*
    - partly calcified tumor of 3 cm in size in the left dorsalcosto-phrenic angle, left hemidiaphragm thickened(Fig.3)
    - left kidney: enlarged, calices of the upper pole and renal pelvis enlarged, nephrolithiasis in the upper pole; normal parenchyma in the lower pole (Fig. 4)
    - no visible connection between the lesion in the kidney and in the lung
i. *Radioisotopic Imaging (Tc 99m - MAG-3):* normal total renal clearance; right renal unit with 70%of total renal function; left renal unit with 30% of total renal function
j. *Bronchoscopy (including bronchio-alveolar lavage, trans-bronchial biopsy and cytology with the covered brush)*
    - no visible tumor, no tuberculous cavity
    - Cytology and biopsy: chronic alveolitis, no neoplastic tissue or specific inflammation
    - Bacteriology: Proteus mirabilis

### DIAGNOSIS AND DISCUSSION

The patient underwent radical left nephrectomy with simultaneous atypical resection of the diseased segment in the caudal lobe of the left lung. The surgical approach was performed through a left thoracoabdominal incision. During the operation extensive perinephric scarring was found. A scarred

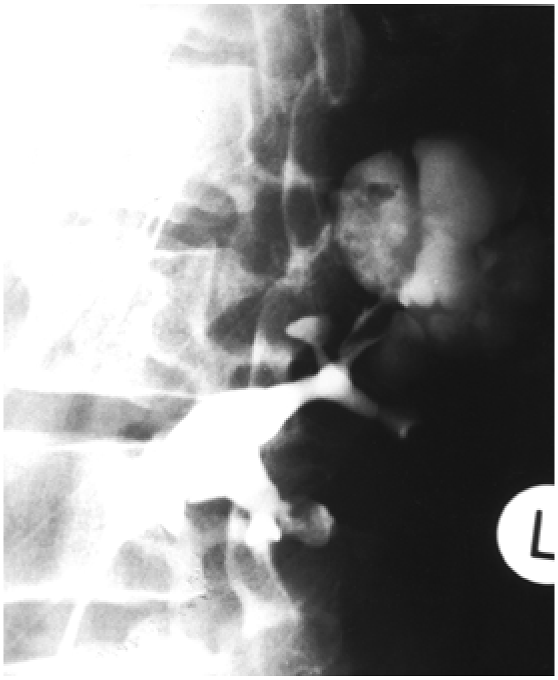

**FIGURE 2**

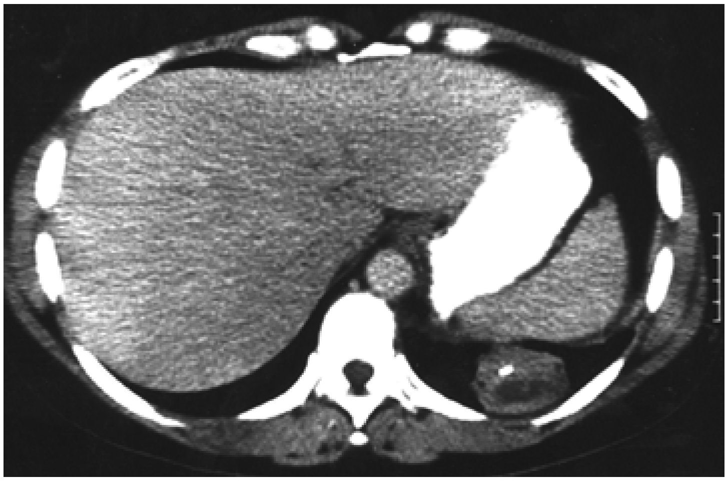

**FIGURE 3**. Mass in the caudal lobe of the left lung.

fistulous tract was identified, which connected the dorsal aspect of the upper pole of the left kidney with the diseased lung segment. On inspection of the specimen after nephrectomy, the renal pelvis and calices were filled with pus and renal stones. Histopathological analysis showed the presence of xanthogranulomatous pyelonephritis (XPGN) of the left kidney which caused the formation of a nephrobronchial fistulous tract. The nephrobronchial fistula was the cause of the recurrent bronchopneumonias. Postoperatively the patient fully recovered and is free of recurrence of pneumonia for 24 months.

Nephro-bronchial fistula is a rare condition. The pulmonary symptoms can be much more impressive than the underlying urological disease. Generally, paranephritic abscess is considered to be the most common etiology of a nephro-bronchial fistula[1]. Nephro-bronchial fistula caused by xanthogranulomatous pyelonephritis (XPGN) is even less common; a medline search resulted in 9 cases reported since 1966.

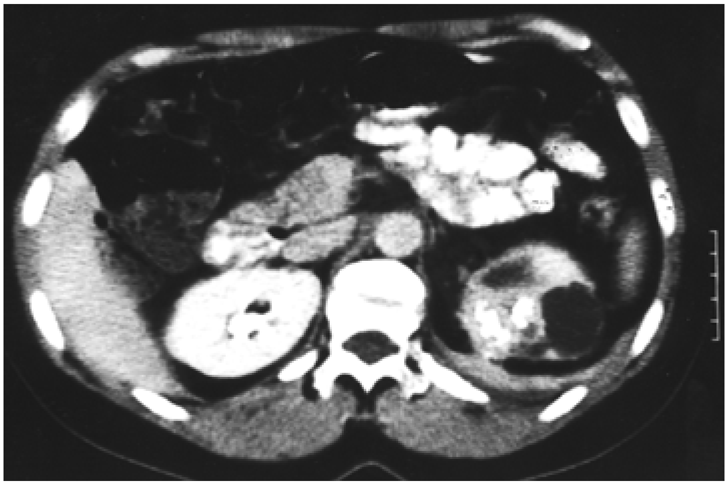

**FIGURE 4**. Mass in the upper pole of the left kidney.

XPGN is an aggressive subtype of common chronic pyelonephritis. It is usually found unilaterally and is three times as common in females than in males. Generally, the diseased kidney is enlarged and deformed by extensive scarring. Renal stones, infection with E. coli and Proteus mirabilis, and urinary obstruction are common. The inflammation often extends into the perinephric space and can be confused with neoplastic infiltration on CT scans or macroscopically. The etiology of the disease remains unclear. Until today, it can be difficult to differentiate XPGN from other renal masses preoperatively. Usually, the final diagnosis is made by the pathologist after nephrectomy. Fistulas caused by XPGN are uncommon, but it has been reported in lung, the spleen, the colon, the duodenum and the skin[2]. There have been reported cases of urothelial cell carcinomas and XPGN in the same kidney[3].

In the case presented, surgical resection was the appropriate treatment. We chose radical nephrectomy because the contralateral renal unit had 70% of the total renal function. The diagnosis was unclear preoperatively. Partial nephrectomies in XPGN have been successfully performed, and in retrospect, an upper pole resection of the left kidney might also have been appropriate in the case presented here.

### REFERENCES

1. Rubin, S. A. and Morettin, L. B.: Nephrobronchial Fistula: an uncommon manifestation of inflammatory renal disease, J. Urol. 127, 103-105, 1982.
2. Chuang, C.K., Lai, M. K., Chang, P. L., Huang, M. H., Chu, S. H., Wu, C.J. and Wu, H. R.: Xanthogranulomatous pyelonephritis: experience in 36 cases. J. Urol. 147 (2), 333-336, 1992.
3. Siemer, S. W., Humke, U., Kramann, B., Becht, E. and Ziegler, M.: Die Xanthogranulomatöse Pyelonephritis: Eine Übersicht mit Kasuistiken zur Differentialdiagnose und verlaufsorientierten Therapie, Akt. Urol. 25, 200- 207, 1994.

**This article should be referenced as follows:**

Ubrig, B., Roth, S., and Hertle, L. (2004) 54 year-old female with recurrent bronchopneumonia and tumor of the left kidney. *TheScientificWorldJOURNAL* **4 (S1)**, 353–356.

**Handling Editor:**

Anthony Atala, Principle Editor for *Urology* — a domain of *TheScientificWorldJOURNAL*.
